# Retarded Catatonia in a 16-Year-Old Male With Schizo-Obsessive Disorder

**DOI:** 10.1192/j.eurpsy.2025.1107

**Published:** 2025-08-26

**Authors:** E. Bolat, E. Akçay, E. Çöp

**Affiliations:** 1 Child and Adolescent Psychiatry, Ankara Bilkent City Hospital, Ankara, Türkiye

## Abstract

**Introduction:**

Catatonia is a complex neuropsychiatric syndrome characterized by a range of motor, cognitive, affective, and autonomic disturbances. It is often associated with psychotic disorders, mood disorders, and pervasive developmental disorders in children. Despite its potential severity, catatonia can be effectively treated with timely intervention, including the use of benzodiazepines and electroconvulsive therapy (ECT). However, treating catatonia in the context of schizo-obsessive disorder presents significant challenges.

**Objectives:**

The case highlights the importance of early diagnosis and intervention in managing catatonia, as well as the need for more ECT sessions in schizo-obsessive catatonia.

**Methods:**

Clinical case report and brief literature review on schizo-obsessive catatonia was done. Informed consent from the patient’s legal guardians was obtained.

**Results:**

We present a case report of a 16-year-old male from Ankara, living with his family, who is a middle school graduate but could not start high school due to his disorder. He had been followed in our outpatient clinic with a diagnosis of Obsessive Compulsive Disorder for 4 years. The patient presented to the emergency department with agitation, disrobing, attempting to climb out of a window, and experiencing delusions. He was initially diagnosed with psychosis and started on risperidone 1 mg and lorazepam 1 mg.

Five days later, during a follow-up, he exhibited non-compliance with commands, mutism, refusal to eat, and urinating in the living room for the past three days. Examination revealed no eye contact, no verbal communication, and a flexed arm posture, leading to a preliminary diagnosis of catatonia and hospital admission. Physical examination, blood tests, brain imaging, and EEG showed no pathological findings. No substances were detected in urine. Despite increasing lorazepam to 6 mg, catatonia symptoms persisted, leading to the initiation of ECT on the fifth day.

After 20 ECT sessions, catatonia symptoms and psychotic content improved, though obsessions persisted. He was diagnosed with schizo-obsessive disorder and treated with fluvoxamine 200 mg/day, olanzapine 10 mg/day, and clonazepam 4 mg/day, with maintenance ECT ongoing.

**Conclusions:**

This case report highlights the complexity of schizo-obsessive catatonia and the necessity for a multifaceted diagnostic and therapeutic approach. The patient’s journey from an initial diagnosis of Obsessive Compulsive Disorder to the emergence of psychotic and catatonic symptoms underscores the fluidity of psychiatric diagnoses. The significant improvement following multiple, longer ECT sessions underscores the therapy’s potency, particularly in schizo-obsesive catatonia. This case underscores the importance of flexibility in psychiatric treatment, advocating for a tailored approach that evolves with the patient’s symptoms.

**Disclosure of Interest:**

None Declared

